# Endotrophin, a fibroblast matrikine, may be a driver of fibroblast activation in fibro-inflammatory diseases

**DOI:** 10.3389/fmolb.2023.1228232

**Published:** 2023-07-12

**Authors:** Alexander L. Reese-Petersen, Federica Genovese, Lei Zhao, Glen Banks, David A. Gordon, Morten A. Karsdal

**Affiliations:** ^1^ Nordic Bioscience A/S, Herlev, Denmark; ^2^ Bristol Myers Squibb, New York, NJ, United States

**Keywords:** fibrosis, biomarkers, heart failure, collagen, ECM, extracellular matrix, endotrophin

## Abstract

Extracellular matrix proteins harbor signaling domains that once released from the parent molecule can trigger cellular responses. One of these molecules is endotrophin, a type VI collagen derived fragment, whose circulatory levels have been associated to an increased risk of adverse outcome in heart failure with preserved ejection fraction (HFpEF). Here we show that the stimulation of human cardiac fibroblasts by endotrophin upregulates the synthesis of type I collagen, the main interstitial collagen that accumulates in the myocardium during fibrogenesis. These data provide a possible mechanistic explanation for the relation between circulating endotrophin levels and risk of outcome in HFpEF.

## Introduction

The core structure of tissues consists of extracellular matrix (ECM) proteins. These molecules serve important structural and cell-regulating functions. During pathological tissue turnover, an increased amount of ECM proteins accumulate in the affected organs (Karsdal et al., 2017). During fibrogenesis, an increased degradation and formation of ECM proteins leads to the release of new epitopes, some of which signals to cells. The main ECM compartment of the body is the interstitial ECM produced predominantly by fibroblasts, and the basement membrane, produced predominantly by epithelial cells. Collagen signaling research has mainly revolved around NC-1 domain peptides from the basement membrane, of which endostatin is the most well described. These signals are anti-angiogenic and are thus highly important for micro- and macrovascular diseases (Karsdal et al., 2017). While basement membrane signals are well understood, emerging research suggest that fibroblast collagens also have signaling functions and play deleterious roles in pathologies, by driving self-perpetuating signaling advancing fibrogenesis.

It is becoming increasingly difficult to overlook the importance of understanding disease-activated fibroblasts and how they contribute to pathology. In the past decade, an increasing amount of attention has been directed at the molecule derived from type VI collagen, endotrophin (Sun et al., 2014).

Endotrophin has been reported as a biomarker prognostic for outcome in a range of pathologies in clinical settings, and is strongly associated with fibro-inflammatory diseases with an underlying component of fibrosis, including chronic liver- and kidney disease, ischemic heart disease, heart failure and diabetes (Staunstrup et al., 2021), but the disease driving potential remains to be fully understood. Endotrophin may be a causal biomarker, being both part of and driving disease pathogeneses in fibro-inflammatory diseases, by activating multiple pathways known to be involved in facilitating more severe disease, driving fibrosis and inflammation, and metabolic dysregulation in general (Sun et al., 2017; Scherer and Gupta, 2021).

Heart failure with preserved ejection fraction (HFpEF) is a heterogenous clinical syndrome known to be affected by fibrosis and metabolic dysregulation (González et al., 2018; Gargiulo et al., 2020), which increases the risk of adverse outcome. There is a great medical need for circulating biomarkers reflecting tissue fibrosis, to complement currently used techniques for assessing risk and biological abnormalities. Molecular insights into the underlying pathobiological mechanisms driving this syndrome could lead to novel treatment strategies and a better understanding of risk of disease progression.

In a recent paper by Chirinos et al., the association between baseline levels of endotrophin and known outcomes in HFpEF patients was investigated through a series of retrospective analyses in clinical cohorts. It was found that endotrophin levels were increased in HFpEF patients, and independently associated with increased risk of hospitalization for HF and mortality, and that prognostic value was added when analyzed in addition to the MAGGIC risk score and NT-proBNP (Chirinos et al., 2022). His could indicate that endotrophin levels offer novel biological information in relation to HFpEF pathogenesis, especially considering the previously described bioactive processes relevant for risk of outcome in HFpEF patients. Based on these findings in a clinical setting, we hypothesized that endotrophin could be involved in HFpEF pathogenesis by directly stimulating fibroblasts and driving a pro-fibrotic response.

**FIGURE 1:**
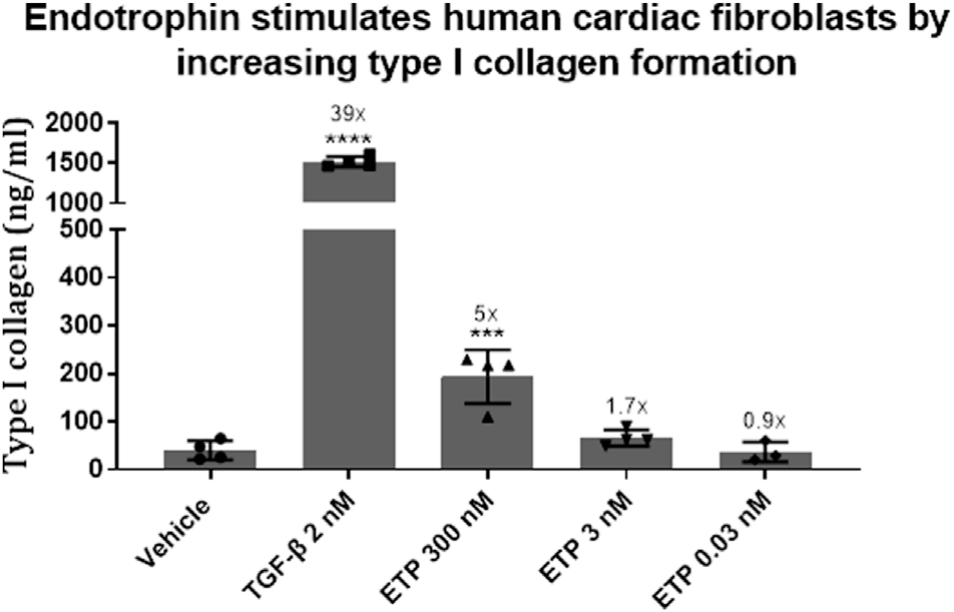
The pro-fibrotic effect of endotrophin was investigated in an *in vitro* cell system using human cardiac fibroblasts (Innoprot, cat. P10453). The cells were treated with 2 nM transforming growth factor-β (TGF-β, R&D Systems, 100-B-010/CF) or either 300, 3 or 0.03 nM endotrophin (custom recombinant expression) for 3 days. Type I collagen formation was assessed in the supernatant by an ELISA developed at Nordic Bioscience (PRO-C1). Differences in type I collagen formation were evaluated using one-way Anova with Dunnett’s multiple comparison test (GraphPad Prism, ver. 9.1.1). Formation of type I collagen was significantly increased by TGF-β (*p* < 0.0001) and endotrophin (300 nM, *p* = 0.0005), resulting in a 39- and 5-fold upregulation in synthesis compared to vehicle.

In order to investigate this potential relationship, we established an *in vitro* model using human primary cardiac ventricular fibroblasts. Upon treating with endotrophin, we found a 5-fold increased expression of type I collagen (Figure 1), the most abundant type of collagen in the myocardium, which decreased in a dose-dependent manner. This could provide a potential mechanism for how endotrophin may drive fibrosis and thus outcome in HFpEF patients by directly inducing fibroblast activation upon its release. An increased level of fibroblast activity and abundance of endotrophin in the myocardium could lead to impaired heart function, contributing to HFpEF pathogenesis and fibrotic diseases in general. Disease-activated fibroblasts may be a part of a fibrogenic endotype, depositing interstitial matrix collagens.

Our experiment is limited by the simplicity of the cell system. Further studies should explore the impact of endotrophin in more complex culture systems and seek to link circulating levels of endotrophin to local upregulation in disease-affected tissues. Moreover, although multiple papers describe expression of endotrophin in various tissues (Lamandé and Bateman, 2018), further studies should seek to investigate disease-specific tissue expression, and further explore the concept of endotrophin being a causal biomarker. Clinical data should also be interpreted in the context of the fact that endotrophin expression likely stems from multiple sources, and further studies are needed to clarify on the pro-fibrotic effects on tissues in fibro-inflammatory diseases.

In summary, we have demonstrated that endotrophin could lead to an increased fibrotic burden in the myocardium by activating and upregulating type I collagen synthesis by cardiac fibroblasts. This is consistent with the opinion in the HFpEF field, and other fibro-inflammatory pathologies, that an increased fibrotic burden is associated with an overall increased risk of outcome. Our data provides potential mechanistic insight in a mechanism of action for endotrophin in relation to increased risk of outcome in HFpEF patients and patients suffering from fibro-inflammatory diseases in general .

Data is presented as means with error bars representing the standard deviation. Numbers above bars indicate -fold upregulation compared to vehicle. TGF-β = transforming growth factor-β; ETP = endotrophin. ****, *p* < 0.0001; ***, *p* = 0.0005.

## Data Availability

The raw data supporting the conclusions of this article will be made available by the authors, without undue reservation.

## References

[B1] Chirinos J. A. Zhao L. Reese-Petersen A. L. Cohen J. B. Genovese F. Richards A. M. (2022). Endotrophic, a collagen VI formation–derived peptide, in heart failure. NEJM Evid. 1, 1–12. 10.1056/evidoa2200091 PMC10465122 37645406

[B2] Gargiulo P. Marsico F. Renga F. Dell’Aversana S. Esposito I. Marciano C. (2020). The metabolic syndrome in heart failure: Insights to specific mechanisms. Heart Fail Rev. 25, 1–7. 10.1007/s10741-019-09838-6 31414215

[B3] González A. Schelbert E. B. Díez J. Butler J. (2018). Myocardial interstitial fibrosis in heart failure: Biological and translational perspectives. J. Am. Coll. Cardiol. 71, 1696–1706. 10.1016/j.jacc.2018.02.021 29650126

[B4] Karsdal M. A. Nielsen S. H. Leeming D. J. Langholm L. L. Nielsen M. J. Manon-Jensen T. (2017). The good and the bad collagens of fibrosis – their role in signaling and organ function. Adv. Drug Deliv. Rev. 121, 43–56. 10.1016/j.addr.2017.07.014 28736303

[B5] Lamandé S. R. Bateman J. F. (2018). Collagen VI disorders: Insights on form and function in the extracellular matrix and beyond. Matrix Biol. 71–72, 348–367. 10.1016/j.matbio.2017.12.008 29277723

[B6] Scherer P. E. Gupta O. T. (2021). Endotrophic: Nominated for best supporting actor in the fibro-inflammatory saga. EBioMedicine 69, 103447. 10.1016/j.ebiom.2021.103447 34153872 PMC8220587

[B7] Staunstrup L. M. Bager C. L. Frederiksen P. Helge J. W. Brunak S. Christiansen C. (2021). Endotrophic is associated with chronic multimorbidity and all-cause mortality in a cohort of elderly women. EBioMedicine 68, 103391. [Internet]. 10.1016/j.ebiom.2021.103391 34044221 PMC8167215

[B8] Sun K. Park J. Gupta O. T. Holland W. L. Auerbach P. Zhang N. (2014). Endotrophic triggers adipose tissue fibrosis and metabolic dysfunction. Nat. Commun. 5, 3485. [Internet]. 10.1038/ncomms4485 24647224 PMC4076823

[B9] Sun K. Park J. Kim M. Scherer P. E. (2017). Endotrophic, a multifaceted player in metabolic dysregulation and cancer progression, is a predictive biomarker for the response to PPARγ agonist treatment. Diabetologia 60, 24–29. [Internet]. 10.1007/s00125-016-4130-1 27717959 PMC5136306

